# Pre-Competition Weight Loss Models in Taekwondo: Identification, Characteristics and Risk of Dehydration

**DOI:** 10.3390/nu12092793

**Published:** 2020-09-12

**Authors:** Katarzyna Janiszewska, Katarzyna E. Przybyłowicz

**Affiliations:** Department of Human Nutrition, Faculty of Food Science, University of Warmia and Mazury in Olsztyn, Słoneczna 45F Str., 10–718 Olsztyn, Poland; katarzyna.przybylowicz@uwm.edu.pl

**Keywords:** martial arts, combat sports, weight loss, body weight, body weight changes, dehydration, young adult, cluster analysis

## Abstract

Athletes use different combinations of weight loss methods during competition preparation. The aim of this study was to identify and characterize pre-competition weight loss models, which describe these combinations. The second aim was to determine if any existing model pose a higher risk of severe dehydration and whether any of the models could be continued as a lower-risk option. The third aim was to explore whether athletes who used different weight management strategies could be differentiated based on age, sex, training experience or anthropometric parameters. Study participants were randomly selected from Olympic taekwondo competitors and 192 athletes were enrolled. Active (47% weight-reducing athletes), passive (31%) and extreme (22%) models have been described. In the extreme model, athletes combined the highest number of different weight loss methods (3.9 ± 0.9 methods vs. 2.4 ± 0.9 in active and 1.5 ± 0.6 in passive), reduced significantly more body mass than others (6.7 ± 3.5% body mass vs. 4.3 ± 1.9% and 4.5 ± 2.4%; *p* < 0.01) and all of them used methods with the highest risk of severe dehydration. The active and passive models could be continued as a lower-risk option, if athletes do not combine dehydrating methods and do not prolong the low energy availability phase. The extreme model carried the highest risk of severe dehydration. Every fifth weight-reducing taekwondo athlete may have been exposed to the adverse effects of acute weight loss. Taekwondo athletes, regardless of age, sex, training experience and anthropometric parameters, lose weight before the competition and those characteristics do not differentiate them between models.

## 1. Introduction

Weight management and body composition adjustments have an impact on athlete’s performance, and they are fundamental aspects of preparation for competition [1]. Nonetheless, some of the weight regulation habits among combat sports athletes, such as rapid weight loss, have a different rationale than to improve one’s athletic skills. The common practice is to lose significant body mass in the days and weeks leading up to being weighed. Athletes aim to compete at the lowest weight class in which they believe to derive a competitive advantage. By doing so, athletes believe that they may not have to face a much larger opponent and gain a strength advantage over a smaller opponent, thus increasing the potential of success [2].

In studies of taekwondo [3] which is a striking sport, pre-competition rapid weight loss did not affect competitive success. It was confirmed only in grappling sports-wrestling [4] and judo [5]. Pieter and colleagues [6] identified that in the case of senior national taekwondo athlete’s height, not weight, was one of the most significant anthropometric factors contributing to match outcomes. Thus, the possible explanation for undertaking weight loss in taekwondo is the socio-cultural and mental component of pre-competition preparation, as previously suggested by Petterson et al. [7]. According to their study, many athletes engaging in rapid weight loss report increased feelings of self-confidence, focus, discipline and professionalism among other attributes [7]. Pre-competition weight loss is often seen as a key part of combat sport.

Pre-competition weight loss is common among Olympic taekwondo athletes. Studies conducted in different countries confirm that most of athletes reduce body mass before a competition and the prevalence of weight losses practices ranges between 63.3% to 88.9% [8,9]. Our preliminary study was consistent with these results and confirmed that 76% of Polish taekwondo athletes undertake pre-competition weight loss [10]. Our study also confirmed that taekwondo athletes use rapid weight loss methods to achieve their weight loss goals.

When rapid weight loss techniques are used, body mass is lost mainly from lean mass (body water and muscle substrates) and gastrointestinal contents. Only a small amount is lost from body fat [11]. Given the amount of water that is stored in the human body and the rapid speed in which it can be manipulated in comparison to other contributors to body mass, the primary focus of the rapid weight loss strategies is body hydration manipulation [11]. Reljic et al. [12] confirmed that habitual rapid weight loss within a few days before boxing competition was achieved nearly exclusively by dehydration. There are three dehydrating strategies that are commonly used among combat sports: fluid restriction, ‘unlocking’ of bound body fluid, e.g., from glycogen stores and additional fluid loss via respiration, urination and sweating [11]. The facilitation of body fluid loss via sweating can include active (exercise-induced) or passive (exposure to hot environment, e.g., sauna) strategies.

Rapid weight loss, even when achieved due to dehydration, if sensibly implemented and with an adequate recovery plan, can be used to optimize competitiveness through mental performance [13]. However, in the absence of these characteristics, it can impair performance and health [14,15]. According to publicized reports of the harmful (and even fatal) effects of pre-competition weight loss, the most dangerous health-related consequences of rapid weight loss are attributed to severe dehydration [16,17,18,19]. Detailed discussion on the performance and health effects of different pre-competition weight loss methods can be found in previous reviews [7,11,15].

Dehydration during pre-competition period can pose an acute risk to athletes’ health, but it can also increase the risk of injury during competition. Dehydrated athletes hope to rehydrate and restore electrolytes in the short time between the official weigh-in and start of the competition. The greater the water deficit, the longer it takes to replenish it [20]. There is less than 20 h for recuperation in taekwondo and this time may not be sufficient to fully rehydrate after the pre-competition weigh-in. In other combat sports, despite ad libitum fluid ingestion, measurements taken 22 or 24 h after the recovery-rehydration period showed signs of dehydration [20,21]. Petterson and Berg [22] proved that even the higher intake of water, both from fluids and food, on the evening preceding the taekwondo competition did not prevent hypohydration on the morning of a competition. Consequently, a significant proportion of taekwondo athletes were not successfully rehydrating and subsequently were competing in a dehydrated state [21,22].

Artioli et al. [23] advocate that rapid weight loss should be banned from combat sports, but the Olympic taekwondo governing bodies do not restrict or control the use of any weight loss method (besides complying with the World Anti-Doping Agency’s recommendations). The only rule, according to the World Taekwondo Federation, which should limit using some of the rapid weight loss methods, is random weigh-in maximum 2 h before the start of the competition. There is only plus 5% tolerance of the upper limit of contestant’s weight category [24]. Nonetheless, this situation allows for free choice of weight loss methods during the process of competition preparation.

Although it was suggested that the differences between gradual body mass adjustments and rapid weight loss are quite clear [23], it is difficult to draw a borderline between them. The reason is that athletes do not use only one method of weight loss. Numerous studies have examined the weight-loss habits in combat sports and the consistent finding was the use of a combination of different techniques during the process [9,25] although no data was available on which methods were most commonly combined.

According to two studies comparing the weight loss management strategies of male and female taekwondo athletes, the prevalence of rapid weight loss and the use of weight loss methods did not differ significantly [9,26]. To our knowledge, there are no available data on the between-sexes comparisons of the number of methods usually combined.

The questionnaires designed to assess weight loss habits among wrestlers [2,27,28] and judo athletes [29] did not aim to analyze the aspect of the simultaneous use of weight loss methods. Therefore, we decided to address this gap and to analyze the combinations of methods. The aim was to use statistical analysis to separate groups of athletes according to similarities and differences of their pre-competition weight-loss behaviors by identifying and characterizing weight loss models. The second aim of this study was to determine if any of the existing models pose a higher risk of severe dehydration and whether any of the existing models could be potentially continued as the lower-risk pre-competition preparation ritual. The third aim was to explore whether athletes who used different weight management strategies could be differentiated by age, sex, training experience or anthropometric parameters.

## 2. Materials and Methods

### 2.1. Participants

Study participants were randomly recruited among contestants of the Polish Taekwondo Championships. The eligibility criteria to be qualified by the Polish Olympic Taekwondo Association (PZTO) for participation in the championships were as follows: age-appropriate for the age category—cadet (12–14 y.o.), junior (15–17 y.o.) and youth (18–21 y.o.), competitor’s license released by PZTO, required taekwondo degree and medical permission to participate in the competition. All players or their legal guardians provided informed written consent. All weight divisions were qualified to participate in this study.

There were 378 competitors qualified as a study population of Polish Taekwondo Championships competitors (23.5% cadets, 42.7% juniors, 33.8% youth). The minimal sample size was determined for a finite population with an unknown size of the fraction of people with a given trait, calculated with a confidence level of 95% and margin of error of 5%. The calculated minimal sample was 191 athletes. 

Study participants were randomly selected from the stratified list of competitors grouped by age category. The share of athletes’ age categories in the final sample was influenced by a lack of consent to participate in this study from the legal guardians of youngest athletes. Only 13 consents were obtained from cadets (6.8% of the final sample). The minimal sample was filled by other randomly selected athletes from juniors (91 athletes; 47.4% of the final sample) and youths (88 athletes; 45.8% of the final sample) and a total of 192 athletes were enrolled.

The ethical approval for the study procedures (number 20/2010) was granted by the Bioethics Committee of the University of Warmia and Mazury in Olsztyn, Poland.

### 2.2. Data Collection

This study was based on cross-sectional design and consisting of a survey and anthropometric measurements.

The validated questionnaire was used to collect the data on pre-competition weight loss and the use of weight loss methods (supplementary file S1). It consisted of questions relating to athletes’ characteristics: age, sex, number of training years and pre-competition weight loss habits during last season: usage of methods, the greatest weight loss and length of typical weight-loss period. Participants were asked to choose the most common weight-loss methods that they usually combine while losing weight before the competition.

We conducted a repeatability (test-retest) and context validity pilot study on 35 athletes from the target group during the validation process. High repeatability of the method was confirmed (continuous values correlation *r* = 0.71–0.96 and relative error 0.3–10.7%; average matching result for dichotomous dependent variables was 94.6%).

During the research session, the participants received coded self-completion questionnaires. The weight and height of participants were measured and coded in line with the questionnaires. The research sessions were conducted in competition venues, on the day of official weighing (the day before a competition) or on the day of competition.

### 2.3. Statistical Analysis

#### 2.3.1. Weight Loss Methods

To compare the use of weight loss methods between sexes, we have applied χ^2^ Pearson test with additional analysis of the phi coefficient (a measure of association for variables in χ^2^ Pearson test). To compare the distribution of the variables between sexes, Mann–Whitney U test was applied.

#### 2.3.2. Identification of Weight Loss Models

The first step to explore the data was the hierarchical clustering. It was used to assess whether groups of athletes that have similar weight-loss patterns and how many such groups (clusters) could be differentiated. To determine the number of clusters we used agglomeration method of complete linkage based on Euclidean distances. Based on the dendrogram, we decided to group athletes into 3 clusters. It was the only a priori assumption regarding the grouping of athletes in the second step of analysis. There have been no prior assumptions regarding the combination of weight loss methods used by athletes.

**Figure 1:**
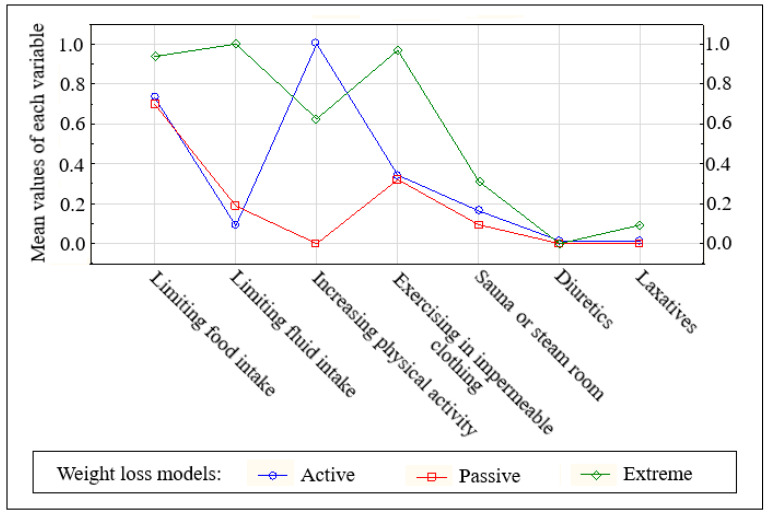
K-means clustering results-graph illustrating mean values of each variable (dimension) that discriminates the three weight loss models. Variables: use of weight loss methods (did not use = 0, used = 1). The supplementary Table S1 presents percentage of athletes using individual weight loss methods in the identified weight loss models.

The second step was to identify three weight loss models (clusters) using a non-hierarchical k-mean cluster analysis method [30]. Input variables were the data on using seven weight loss methods among weight-reducing athletes (Figure 1), with dichotomous response range (no = did not use = 0, yes = used = 1).

The sample size included in this multidimensional analysis (144 athletes who lost weight before a competition) was more than 20 times larger than the number of input variables (7). This value has suggested a very high level of statistical inference from the cluster analysis.

The variables “increasing physical activity” and “limiting fluid intake” were the main criterion for cluster distinction (respectively F = 295.1 and F = 105.7). Based on the analysis of weight loss patterns, athletes were separated into active, passive and extreme models.

Characteristics of weight loss process in weight loss models were compared by Kruskal–Wallis ANOVA test with post-hoc test when the difference was significant.

#### 2.3.3. Risk of Dehydration

Severity index (SI) was used [31] to assess the risk of dehydration resulting from the use of weight loss methods. SI describes the risk of severe dehydration on a 3-point scale. Methods bearing the highest risk of serious dehydration are scored with 3 points. The following SI values were adopted in this study: SI = 1—limiting food intake, increasing physical activity; SI = 2—limiting fluid intake; SI = 3—exercising in impermeable clothing, using sauna, laxatives, diuretics, and vomiting.

For each athlete, we determined the average SI value of the methods used. Then the average SI value of each cluster was calculated. Besides, the share of the following groups among all study participants and identified weight-loss models was determined:

- Athletes using only the methods with the lowest value of SI index (SI = 1, SI ≠ 2, SI ≠ 3);
- Athletes using a method with the value of SI = 2, that is limiting fluid intake, without methods with the highest SI value (SI = 1 and SI = 2, SI ≠ 3);
- Athletes using at least one method with the highest SI value (SI = 1 or SI = 2 and SI = 3).

To compare the severity index of weight loss methods in weight loss models we have applied χ^2^ Pearson test with additional analysis of C-Pearson’s contingency coefficient. The Kruskal–Wallis ANOVA test was used to compare the distribution of the variables between models, with post-hoc test when the difference was significant

#### 2.3.4. Characteristics of Athletes with Different Weight Loss Strategies

The percentile values and standard deviation index (Z-score) of height, weight and body mass index (BMI) were calculated according to references for growth and nutritional status assessment in Polish children and adolescents [32]. For participants over 18 years of age, the same development standards as for 18-year-olds were adopted. We determined the percentage of athletes with very low (Z-score < −2SD) and low (<10 percentile, Z-score <−1SD) weight and BMI indicating the risk of malnutrition [33]. Mean values of anthropometric parameters and percentages of athletes with the risk of malnutrition were compared between groups of athletes who did not lose weight and athletes losing weight by different models.

Several comparison analyses were performed. For group comparisons with categorical data, we have applied χ^2^ Pearson test. Kruskal–Wallis ANOVA test was used to compare the distribution of the variables between models.

The statistical analyses were conducted with Statistica PL software (Statistica, version 13 PL, TIBCO Software Inc., Palo Alto, Santa Clara, CA, United States).

## 3. Results

### 3.1. Weight Loss Methods

The most commonly used weight loss methods were limiting food intake (60%) and increasing physical activity (45%; Table 1). Differences between male and female athletes were reported in the use of saunas with a predominance of use in men (19% vs. 9%; *p* < 0.05), however, the strength of this association was low (Φ = 0.14). None of the male participants used pharmaceuticals and no one from the study group induced vomiting as a method of rapid weight loss. 

The majority (56.8%) of weight-reducing athletes used more than one weight-loss method simultaneously. The highest percentage of male (20.9%) and female (27.7%) respondents used two weight loss methods simultaneously

### 3.2. Weight Loss Models—Identification and Characteristics

The use of weight loss methods and the number of combined methods were not significantly different between sexes. There was no need to separate male and female weight loss models. Consequently, they were extracted from the whole weight-reducing study sample (*n* = 144).

#### 3.2.1. Identification of Weight Loss Models

Three clusters have been created to describe models of pre-competition weight loss: active, passive and extreme (Figure 1). The athletes were assigned to each cluster (model) by similarities in combining weight loss methods. 

The biggest cluster (47.0% of weight-reducing athletes, 36.7% of all athletes), called the active model, grouped individuals who increased physical activity, very often combined with limited food intake. Unlike in the active model, athletes from the passive model (31.3% of weight-reducing athletes, 24.7% of the total sample) did not increase physical activity before the competition. They limited food intake and some of them used impermeable suits during exercise. The third cluster, the extreme model, grouped 21.2% of weight-reducing athletes (17.5% of the total sample) who limited fluid and food intake and used impermeable suits during exercise. One-third of them used the sauna and some of them also laxatives.

#### 3.2.2. Characteristics of the Weight-Loss Process in Different Models

The weight loss models separated athletes who combined a different number of weight loss methods (Table 2). Athletes from the passive model used the smallest number of weight loss methods, athletes from the extreme one used simultaneously four different techniques.

Time taken for weight loss was similar in all three models. Athletes from the extreme model reduced significantly more body mass than athletes who used the active and passive models.

### 3.3. Weight Loss Models and Dehydration Risk

The percentage of athletes using only the methods with the lowest risk of dehydration (SI = 1; Table 3), i.e., reduced food consumption and increased physical activity, was 34.7%. Neither of them was classified in the extreme model because all athletes in the extreme model used at least one method of the highest risk of dehydration (S = 3). The percentage of athletes using the highest SI methods in the remaining models was significantly lower than in the extreme one and below the half of the group (41.8% and 42.2%). Thus, the mean SI of the methods from the extreme model was significantly higher than in other models. The lowest values of the mean SI were observed among athletes from the active model.

### 3.4. Characteristics of Athletes with Different Weight Management Strategies

Weight management strategies did not differentiate the athletes by mean age, sex, training experience, height or BMI (Table 4). Significantly more athletes from the youth category did not lose weight during the last season compared to the juniors (31.8% vs. 17.6%). A reverse proportion was found between the age categories in the passive model (18.0% youth vs. 29.7% juniors).

All weight divisions were qualified to analyze anthropometric parameters, even heavyweight—the highest category without an upper weight limit (also called ‘open’). Analysis of development standards of weight-to-age showed that the mean percentile of athletes who did not lose weight was higher than those who were classified as the extreme model. There were no further differences between the mean values of anthropometric parameters between the groups.

The highest share of athletes with the risk of malnutrition, expressed as BMI-to-age <10 percentile, was found in the passive model, the lowest in the extreme model. When the same groups were compared by the share of athletes with the risk of malnutrition, expressed as z-score <−1SD, the differences were not significant.

## 4. Discussion

### 4.1. Weight Loss Methods

The most common weight loss strategy was maintaining a negative energy balance (through limiting food intake and/or increasing physical activity). Similar conclusions were drawn from studies involving athletes from other combat sports: box, Brazilian jiu-jitsu, wrestling, judo, Muay Thai/kickboxing and mixed martial arts [2,34,35,36,37,38].

Our results are consistent with conclusions made by Reale et al. [12] that sweating is the most common method of dehydration undertaken by combat sports athletes. More than one-third of our study sample used impermeable clothing (nylon or ‘sauna suits’) during exercise in order to induce dehydration. Vigorous exercise and dehydration increase body temperature. The use of impermeable suits reduces evaporation and heat loss by convection, which results in a further increase in body temperature [17]. Thus, this method is a combination of active and passive sweating and carries a high risk of severe dehydration (SI = 3) [31]. Although the sex difference was not significant, the use of diuretics, laxatives-potentially hazardous and banned methods of rapid weight loss, that also carry the risk of severe dehydration were present only among female athletes. According to Artioli et al. [39], the percentage of judokas (male and female) using pharmacological agents in order to reduce weight was significantly higher—20% of athletes used laxatives and 16% diuretics. Due to the potential side effects of using such extreme methods, attention should be paid to discouraging athletes from using them. It is important to emphasize that diuretics are used despite their inclusion on the World Anti-Doping Agency’s List of Prohibited Substances [40] and are responsible for the reported cases of doping in combat sports [41,42].

### 4.2. Weight Loss Models—Identification and Characteristics

The active model accumulated almost half of the weight-reducing athletes (46.5%), considerably more than the remaining two models. Weight loss methods commonly used in this model are the core of the recommended gradual weight loss concept [43]. A combination of methods similar to the active model: reduced energy intake, increased exercise and dehydration but with a magnitude of 5% reduction of body mass resulted in a 54% decrease in muscle glycogen concentration [44]. Glycogen stores are a very important source of energy during the taekwondo tournament [45,46]. After a weight reduction process, restoring glycogen to baseline can last up to 72 h [47]. Official general weigh-in of taekwondo athletes is organized on the day preceding the start of the tournament, that is, athletes have less than 20 h for regeneration [24]. Therefore, in the case of significantly reduced glycogen stores after the weight loss process, the athlete’s ability to exercise during the tournament may still be impaired. Nonetheless, the glycogen depletion does not pose a direct health risk to an athlete.

The difference between the active and passive models was undertaking additional physical activity. In terms of analyzing the active model’s safety, we need to consider that athletes at a highly competitive level have already a high training load. If weight cycling is repeated multiple times during the season, it can become the risk factor of overtraining or developing Relative energy deficiency in sport (RED-S). RED-S leads to long-term negative health consequences, which could be especially dangerous for young athletes [48]. In the passive model, the alternative to increased physical activity could be the reduction of food intake or using rapid weight loss methods. Metabolic responses to low energy availability achieved by diet or exercise are different as concluded by Papageorgiou et al. [49]. Lower energy availability during three days of dietary energy restriction in active women resulted in a significant decrease in bone formation whereas low energy availability achieved through exercise energy expenditure did not significantly influence bone metabolism. Bone formation is an important aspect of bone turnover, especially during adolescence, and prolonging this weight loss phase can affect the athletes’ bone health.

Even though 34.3% of athletes in the active model used impermeable clothing during training, almost all of them (91%) rehydrated normally—only 9% of them limited fluid intake. The passive model was similar in this aspect—37.8% of athletes in the active model used impermeable clothing but most of the athletes (77.8%) rehydrated as usual and did not limit fluid intake. The situation was different in Extreme model, where 96.9% of athletes used impermeable clothing. However, all of them also limited fluid intake. The most common weight loss method in the passive model was limiting food intake, and athletes rarely used other methods simultaneously. It can be assumed that their weight loss pattern was mostly based on gradual weight loss achieved by negative energy balance.

Athletes who were classified as the extreme model combined several weight loss methods. Almost all of them limited fluid and food intake and trained in impermeable clothing. When considering the number of weight loss methods and the amount of body mass lost as means of aggressiveness of rapid weight loss pattern [29], we can conclude that the extreme model is the most aggressive one. The least aggressive model is the passive one. In this model, there was no incidence of using three (increasing physical activity, using laxatives and diuretics) out of seven analyzed methods. According to Artioli et al. [29], using laxatives and diuretics double the risk for health, compared to other methods, and highly increase the aggressiveness of weight loss pattern.

### 4.3. Weight Loss Models and Dehydration Risk

Athletes who were classified as the extreme model were most likely to induce dehydration by combining several acute weight loss methods. They also declared reducing the largest amount of body mass, however, the time of their typical weight loss was not longer than in other models. This confirms the assumption that in this model athletes use the most aggressive pattern of rapid weight loss, which is mostly based on dehydration. Case reports of sudden deaths of three wrestlers [17] described the combination of methods similar to the extreme model. All three athletes followed the combination of restricted food and fluid intake, wearing vapor-impermeable suits under cotton warm-up suits and exercised vigorously in hot environments. Another reported case of death of an 18-year old female Muay-Thai athlete during her pre-competition preparation was also connected to combining the dehydration methods [50]. The athlete used: water-loading, reducing water intake, running in a rubber sweatsuit, and taking hot, salty baths. Similarities in the combination of methods with the extreme model suggest that using this model can be life-threatening.

It needs to be emphasized that the key factor to describe the real risk of choosing the extreme model is the magnitude of weight loss due to dehydration. Mild dehydration is usually not risky for athletes. They are used to being dehydrated due to conditions they face during everyday trainings. Caldwell et al. [51] suggested that the effect of body fluid loss on performance might be less detrimental if the deficit in body fluid develops slowly. Furthermore, Smith et al. [52] found no relationship between changes in plasma volume and boxing-related tasks in collegiate boxers who had reduced their body mass by 3% to 4% within a few hours by excessive sweat loss. Authors suggested that some athletes might be predisposed to cope effectively with the negative effects of dehydration, and sporting performance might remain unaffected. On the other side, a fatal case study described by Murugappan et al. [19] warns that an athlete’s individual intrinsic factors can have a profound influence on reduced dehydration tolerance, some of which may remain hidden until the accident.

### 4.4. Weight Management Strategies and Athletes’ Characteristics

The weight loss models failed to differentiate athletes by age, sex and training experience. Even younger, less experienced athletes followed each of the weight loss models—including the extreme one. Despite the small number of the youngest participants, our data confirmed that the weight loss behaviors were present in every age category, starting from 12-year-olds, as also reported by de Silva Santos et al. [9] and Berkovich et al. [53]. As mentioned by de Silva Santos et al. [9] children and adolescents may face additional health risks of weight loss compared to their adult counterparts and should not be encouraged to do so.

Analysis of the athletes’ share from different age categories in weight loss models has shown that older athletes didn’t use the passive model as prevalent as younger ones. It seems that with age athletes tend to resign from the Passive model in favor of not losing weight at all. According to Artioli et al. [29] younger athletes chose more aggressive weight loss patterns, but the percentage of athletes in the extreme model was equal in all age categories in our study.

Among the weight-losing athletes, we have identified athletes at risk of malnutrition. As recommended by Ad Hoc Research Working Group on Body Composition, Health and Performance, under the auspices of the International Olympic Committee Medical Commission [54], their body composition should be assessed before the weight loss was started. Body composition was not measured during this study but all the athletes falling into at least one of ‘at risk of malnutrition’ parameters were below 18 years old. Given the biological changes occurring in athletes during adolescence, it is essential to remember that those athletes need special considerations when the weight category is chosen. In the case of non-controlled weight loss during the growth period, the long-term detrimental effects can occur [55].

There were some limitations to the current study. First, the cross-sectional design of the study based on athletes’ declarations. However, the cross-sectional design was needed to include a representative sample of the population, which was the major strength of the study. The second limitation was the lack of additional information relating to dietary weight loss practices and the participants’ body composition. Such information could help to assess if there were any health risks associated with the most common method of limiting food intake. The incidents of fasting or the risk of athletes’ malnutrition or RED-S have not been identified. We were also unable to assess whether there was any reduction in the consumption of certain nutrients, e.g., carbohydrates to dehydrate or fiber to manipulate gut contents.

However, the study’s strength was the use of a validated questionnaire and a strong statistical method for pre-competition models identification and characterization. This body of work adds value to the existing literature on weight loss in combat sports by differentiating the weight loss models, as a continuum of studies of single methods recognition. Such designation reflects the real picture of combat sports athletes’ behaviors. It better differentiates those who might be at higher health risk.

## 5. Conclusions

Olympic taekwondo athletes combine weight loss methods according to three models: active, passive and extreme. Most weight-reducing taekwondo athletes, who followed the active or passive model, can continue their lower-risk pre-competition preparation ritual if they do not combine dehydrating methods and do not prolong the low energy availability phase.

Athletes from the extreme model by combining several acute dehydration methods, had a potentially high risk of severe dehydration. It means that every fifth weight-reducing taekwondo athlete, who was classified to the extreme model, may have been exposed to the adverse effects of acute weight loss and even loss of health or life. Taekwondo athletes regardless of age, sex, training experience and anthropometric parameters lose weight before the competition and those characteristics do not differentiate them between models. Even the youngest, less experienced athletes, followed the extreme model, which could lead to severe dehydration.

### Practical Implications

Weight loss by using an extreme model, which focuses solely on qualifying for a lower weight class despite the lack of evidence of competitive advantage, due to the highest risk of dehydration, should be stopped. Athletes from the extreme model, in order to reduce their health risk, should be identified and become subject to special control and weight management support of the training staff. The younger the athlete, the more the family’s involvement is recommended [54] and the results of this study are the evidence that young athletes are a big part of a population of weight-losing athletes. Bearing in mind the dynamic development of taekwondo as an Olympic sport, there is a need to formulate recommendations and educational programs for coaches, athletes and parents, which will allow them to reorient towards health-centered weight management.

## Figures and Tables

**Table 1:** The use of weight loss methods in study sample [%].

Weight Loss Methods	Total (*n* = 192)	Female (*n* = 101)	Male (*n* = 91)	*p* χ^2^ /Φ/
Limiting food intake	60.4	66.3	42.2	0.077
Increasing physical activity	45.3	49.5	40.7	0.219
Exercising in impermeable clothing	37.0	39.6	34.1	0.427
Limiting fluid intake	25.0	24.8	25.3	0.934
Sauna or steam room	13.5	8.9	18.7	0.048/0.14/
Laxatives	2.1	4.0	0	0.055
Diuretics	0.5	1.0	0	0.341
Vomiting	0	0	0	-
**Number of combined** **methods**				
[x ± SD]	1.8 ± 1.5	1.9 ± 1.5	1.7 ± 1.5	0.320 *
[% in column]				
6	1.0	2.0	0.0	0.617
5	4.2	4.0	4.4	
4	8.3	9.9	6.6	
3	18.8	16.8	20.9	
2	24.5	27.7	20.9	
1	18.2	16.9	19.8	
0 (did not lose weight)	25.0	22.8	27.5	

*p* χ^2^—χ^2^ Pearson test between female and male athletes; Φ—phi coefficient, a measure of association for variables in χ^2^ Pearson test; *—Mann–Whitney U test between female and male athletes.

**Table 2:** Characteristics of weight loss process in weight loss models.

Characteristics	Total (*n* = 144)	Weight Loss Models			*p*
		Active (*n* = 67)	Passive (*n* = 45)	Extreme (*n* = 32)	
Number of combined weight loss methods [x ± SD]	2.3 ± 1.3	2.4 ± 0.9 ^a^	1.5 ± 0.6 ^a^	3.9 ± 0.9 ^a^	0.000
The greatest weight loss [kg; x ± SD]	2.9 ± 1.5	2.6 ± 1.3 ^b1^	2.7 ± 1.5 ^b2^	3.8 ± 1.7 ^b1,b2^	0.009
The greatest weight loss [% body mass; x ± SD]	4.9 ± 2.6	4.3 ± 1.9 ^b1^	4.5 ± 2.4 ^b2^	6.7 ± 3.5 ^b1,b2^	0.015
Length of weight loss period [% in column]					
1–2 days	14.2	16.9	13.6	9.4	0.623 *
3–5 days	5.7	3.1	4.6	12.5	
ca. 1 week	34.8	33.9	36.4	34.4	
ca. 2 weeks	27.7	23.1	34.1	28.1	
ca. 3 weeks	9.2	12.3	6.8	6.3	
ca. month	8.5	10.8	4.6	9.4	

x ± SD—values given as mean and standard deviation; *p*—Kruskal–Wallis ANOVA test between models; *—χ^2^ Pearson test between models; a, b1, b2–statistical difference between models in couple comparison in rows (respectively *p* < 0.001; *p* < 0.01; *p* < 0.01).

**Table 3:** Classification of athletes according to the severity index of weight loss methods in weight loss models [%].

SI of Weight Loss Methods	Total (*n* = 144)	Models			*p*
		Active (*n* = 67)	Passive (*n* = 45)	Extreme (*n* = 32)	
SI = 1 (SI ≠ 2, SI ≠ 3) [% in column]	34.7	49.3 ^a1^	37.8 ^a2^	0.0 ^a1,a2^	0.000 ^#^
SI = 1 and SI = 2 (SI ≠ 3) [% in column]	10.4	9.0	20.0 ^b^	0.0 ^b^	/0.46/
SI = 1 or SI = 2 and SI = 3 [% in column]	54.9	41.8 ^a1^	42.2 ^a2^	100.0 ^a1,a2^	
Mean SI [x ± SD]	1.6 ± 0.6	1.4 ± 0.4 ^a,c^	1.7 ± 0.7 ^b,c^	2.0 ± 0.2 ^a,b^	0.000 *

SI—severity index describing the risk of severe dehydration when using the method; *p*—the significance of the differences between the models; *—Kruskal–Wallis ANOVA test; #—χ^2^ Pearson test; //—C Pearson’s contingency coefficient; x ± SD—values given as mean and standard deviation; SI = 1—athletes using only methods with an index value of 1; SI = 1 and SI = 2—athletes using methods with the value of 2, that is limiting fluid intake, without methods with the value of 3; SI = 1 or SI = 2 and SI = 3—athletes using at least one method with the index value of 3; a, a1, a2, b, c—statistical difference between models in couple comparison (respectively *p* < 0.001; *p* < 0.001; *p* < 0.001; *p* < 0.01; *p* < 0.05).

**Table 4:** Athletes’ characteristics in different weight management strategies.

Characteristics	Total (*n* = 192)	Did not Lose Weight (*n* = 48)	Weight Loss Models			*p*
			Active (*n* = 67)	Passive (*n* = 45)	Extreme (*n* = 32)	
**Age [years;** x ±SD]	17.7 ± 2.1	18.2 ± 2.2	17.5 ± 2.0	17.6 ± 2.0	17.6 ± 2.0	0.319
**Age category** [% in row]						0.333 *
Cadet (*n* = 13)		30.8	38.5	15.4	15.4	
Junior (*n* = 91)		17.6 ^a^	35.2	29.7 ^a^	17.6	
Youth (*n* = 88)		31.8 ^b^	34.1	18.0 ^b^	15.9	
**Sex [% females]**	52.6	47.9	55.2	55.6	50.0	0.837 *
**Training experience [years;** x ± SD]	6.2 ± 2.9	6.7 ± 3.3	5.6 ± 2.6	6.7 ± 2.5	6.1 ± 3.1	0.489
**Height**						
height-to-age [pc; x ± SD]	59.8 ± 29.9	63.8 ± 30.7	60.6 ± 28.1	62.3 ± 31.1	48.9 ± 29.6	0.120
z-score	0.3 ± 1.0	0.5 ± 1.0	0.4 ± 1.0	0.4 ± 1.2	−0.1 ± 1.0	0.053
**Weight**						
weight-to-age [pc; x ± SD]	51.7 ± 28.1	60.9 ± 29.3 ^c^	52.8 ± 27.3	47.2 ± 28.3	41.5 ± 23.9 ^c^	0.017
<10 pc [% in column]	7.5	6.3	7.7	8.9	6.7	0.187 *
z-score [x ± SD]	0.3 ± 1.2	0.7 ± 1.5	0.3 ± 1.0	0.1 ± 1.0	0.0 ± 0.9	0.040
z-score [% in column]						0.115 *
−1SD > z-score > −2SD	10.1	6.3	12.3	13.3	6.7	
z-score < −2SD	1.1	4.2	0.0	0.0	0.0	
**BMI**						
BMI-to-age [pc; x ± SD]	47.7 ± 27.9	55.4 ± 29.5	47.4 ± 27.4	41.7 ± 28.6	45.1 ± 23.6	0.108
<10 pc [% in column]	9.6	8.3	7.7	20.0 ^b^	0.0 ^b^	0.033 *
z-score [x ± SD]	0.1 ± 1.1	0.4 ± 1.3	0.0 ± 1.0	−0.2 ± 0.9	0.0 ± 0.8	0.118
z-score < −1SD [% in column]	14.4	14.6	13.8	20.0	6.7	0.225 *

x ± SD—values given as mean and standard deviation; *p*—Kruskal–Wallis ANOVA test between models; pc—percentile; *—χ^2^ Pearson test between models; a, b, c—statistical difference in couple comparison in row (respectively *p* < 0.001, *p* < 0.01; *p* < 0.05).

## Supplementary Materials

The following are available online at https://www.mdpi.com/2072-6643/12/9/2793/s1, Table S1: Percentage of athletes using individual weight loss methods in the identified weight loss models [%]. File S1: Polish and English version of Pre-competition Weight Loss Questionnaire.
