# Supplementary material for: Pre-Competition Weight Loss Models in Taekwondo: Identification, Characteristics and Risk of Dehydration
Source: Nutrients. 2020 Sep 12;12(9):2793. doi: 10.3390/nu12092793 (PMC7551247; doi:10.3390/nu12092793)
Supplement: Supplementary file 1 [file nutrients-12-02793-s001.zip › nutrients-907124-supplementary/table S1 .docx]

**Table S1.** **Percentage of athletes using individual weight loss methods in the** **identified weight loss models [%]**

| **Weight loss techniques** | **Total**  **(n=144)** | **Models** | | | **p χ2** | **C** |
| --- | --- | --- | --- | --- | --- | --- |
|  |  | **Active**  **(n=67)** | **Passive**  **(n=45)** | **Extreme**  **(n=32)** |  |  |
| Limiting food intake | 80.6 | 73.1^c^ | 82.2 | 93.8^c^ | 0.050 | 0.20 |
| Increasing physical activity | 60.4 | 100.0^a^ | 0^a^ | 62.5^a^ | 0.000 | 0.66 |
| Exercising in impermeable clothing (sauna suits) | 49.3 | 34.3^a1^ | 37.8^a2^ | 96.9^a1,a2^ | 0.000 | 0.45 |
| Limiting fluid intake | 33.3 | 9.0^a1^ | 22.2^a2^ | 100.0^a1,a2^ | 0.000 | 0.61 |
| Sauna | 18.1 | 16.4 | 11.1^c^ | 31.3^c^ | 0.069 | 0.19 |
| Laxatives | 2.8 | 1.5 | 0^c^ | 9.4^c^ | 0.033 | 0.21 |
| Diuretics | 0.7 | 1.5 | 0 | 0 | 0.561 | 0.09 |

p χ^2^ – χ^2^ Pearson test between models; C - Pearson's contingency coefficient; a-a, a1-a1,a2-a2 c-c – statistical difference between models in couple comparison (respectively p<0.001; p<0.001; p<0.001; p<0.05)
