# Supplementary material for: Pre-Competition Weight Loss Models in Taekwondo: Identification, Characteristics and Risk of Dehydration
Source: Nutrients. 2020 Sep 12;12(9):2793. doi: 10.3390/nu12092793 (PMC7551247; doi:10.3390/nu12092793)
Supplement: Supplementary file 1 [file nutrients-12-02793-s001.zip › nutrients-907124-supplementary/file S1.doc]

**[PL]**

**Kwestionariusz dotyczący przedstartowej redukcji masy ciała**

*Wyniki zostaną wykorzystane wyłącznie w celach naukowych.*

PYTANIA OGÓLNE

***1. Data badania***	|_|_| / |_|_| / |_|_|_|_| (dzień/miesiąc/rok)

***2. Data urodzenia***	|_|_| / |_|_| / |_|_|_|_| (dzień/miesiąc/rok)

***3. Płeć***

1. Mężczyzna

2. Kobieta

***4. Od ilu lat trenujesz taekwondo?***

………………………………. lat

***5. W jakiej kategorii wagowej startowałeś/łaś w ostatnim sezonie?***

………………………………. kg

PYTANIA DOTYCZĄCE PRZEDSTARTOWEJ REDUKCJI MASY CIAŁA

***6. Czy w ostatnim sezonie redukowałeś/łaś masę ciała („zrzucałeś/łaś”, „ścinałeś/łaś”, „robiłeś wagę”) przed zawodami?***

1. tak

2. nie	̶˃ *dziękujemy za udział w badaniu, przejdź na pomiary antropometryczne*

***7. Ile czasu przed turniejem zazwyczaj poświęcałeś/łaś na redukcję masy ciała?*** *(wybierz tylko jedną odpowiedź)*

1. 1-2 dni

2. 3-5 dni

3. około tygodnia

4. około 2 tygodni

5. około 3 tygodni

6. około miesiąca

***8. Jaka jest największa ilość kilogramów, które redukowałeś/łaś przed zawodami w ostatnim sezonie?***

………………………………. kg

***9. Które z wymienionych metod zwykle łączysz w procesie odchudzania przed zawodami?*** *(wybierz dowolną ilość odpowiedzi)*

1. ograniczenie spożycia żywności

2. ograniczenie picia płynów

3. zwiększenie aktywności fizycznej (ilości, długości treningów)

4. wypacanie ćwicząc w nieprzepuszczalnej odzieży

5. wypacanie w saunie lub łaźni parowej

6. leki moczopędne (np. Urosept, Furosemid)

7. środki przeczyszczające (np. Xenna)

8. powodowanie wymiotów

9. inne ̶˃ *wymień jakie?* ……………………………………………….

**[EN]**

**Pre-competition Weight Loss Questionnaire**

*The results will be used for research purposes only.*

GENERAL QUESTIONS

***1. Today's date***	|_|_| / |_|_| / |_|_|_|_| (day/month/year)

***2. Date of birth***	|_|_| / |_|_| / |_|_|_|_| (day/month/year)

***3. Sex***

1. Male

2. Female

***4. How many years have you been training taekwondo?***

………………………………. years

***5. What weight category did you fight in last season?***

………………………………. kg

QUESTIONS ON PRE-COMPETITION WEIGHT LOSS

***6. Did you lose weight („cut weight”, „make weight”) before competition during last season?***

1. yes

2. no ̶˃ *thank you for participating in the study, go to anthropometric measurements*

***7. How much time does it usually take you to lose weight before the competition?*** *(choose only one answer)*

1. 1-2 days

2. 3-5 days

3. ca. 1 week

4. ca. 2 weeks

5. ca. 3 weeks

6. ca. month

***8. What is the most weight that you lost last season to make weight before competition?***

………………………………. kg

***9. Which of the following methods do you usually combine during pre-competition weight loss process?*** *(choose all that apply)*

1. Limiting food intake

2. Limiting fluid intake

3. Increasing physical activity (number, length of training sessions)

4. Sweating by exercising in impermeable clothing

5. Sweating in sauna or steam room

6. Diuretics

7. Laxatives

8. Vomiting

9. other ̶˃ *which one?* ……………………………………………….
